# Treatment of anastomotic leak in colorectal surgery by endoluminal vacuum therapy with the VACStent avoiding a stoma - a pilot study

**DOI:** 10.1007/s00423-024-03426-5

**Published:** 2024-07-31

**Authors:** Markus M. Heiss, Jonas Lange, Judith Knievel, Alexander Yohannes, Ulrich Hügle, Arno J. Dormann, Claus F. Eisenberger

**Affiliations:** 1 Department of Abdominal, Tumor, Transplant and Vascular Surgery, Cologne-Merheim Medical Center, Witten/Herdecke University, Ostmerheimer Strasse 200, D-51109 Cologne, Germany; 2 Department of Gastroenterology, Cologne-Holweide and Merheim Medical Center, Cologne, Germany

**Keywords:** Endoscopic treatment by vacuum therapy (EVT), Lower gastrointestinal wall defects, Anastomotic leakage (AL), Colorectal surgery, VACStent, Anus praeter

## Abstract

**Purpose:**

Anastomotic leak (AL) represents the most relevant and devastating complication in colorectal surgery. Endoscopic vacuum therapy (EVT) using the VACStent is regarded as a significant improvement in the treatment of upper gastrointestinal wall defects. The innovative concept of the VACStent was transferred to the lower GI tract, gaining initial experience by investigating safety and efficacy in 12 patients undergoing colorectal resections.

**Methods:**

The pilot study, as part of a German registry, began with 2 patients suffering from AL, who were treated with the VACStent after stoma placement. Subsequently, 6 patients with AL were treated with the VACStent omitting a stoma placement, with a focus on fecal passage and wound healing. Finally, the preemptive anastomotic coverage was investigated in 4 patients with high-risk anastomoses to avoid prophylactic stoma placement.

**Results:**

In total 26 VACStents were placed without problems. The conditioning and drainage function were maintained, and no clogging problems of the sponge cylinder were observed. No relevant clinical VACStent-associated complications were observed; however, in 2 patients, a dislodgement of a VACStent occurred. The 6 patients with AL but without stoma had a median treatment with 3 VACStents per case with a laytime of 17 days, leading to complete wound healing in all cases. The 4 prophylactic VACStent applications were without complications.

**Conclusion:**

The clinical application of the VACStent in the lower GI tract shows that successful treatment of anastomotic colonic leaks and avoidance of creation of an anus praeter is possible.

**Trial registration number:**

Clinicaltrials.gov NCT04884334, date of registration 2021-05-04, retrospectively registered.

## Introduction

AL represents one of the most significant and feared complications of colorectal surgery and has not substantially decreased over the past decades despite advances in surgical techniques [1]. AL is associated with prolonged hospital stays, increased morbidity and reduced survival following cancer resections [2].

The incidence of AL varies from 2 to 39% and is inversely proportional to the distance of the anastomosis from the anal verge [3]. Intraoperative technical problems with the creation of an anastomosis, often indicated by a positive air bubble test, also lead to AL and stoma formation [4].

However, pre-operative prediction of AL and identification of at-risk patients are not accurate, and AL is often diagnosed too late. The management of AL depends on age, comorbidity, and patient stability, often leading to a permanent stoma with a low anastomosis and, in the worst cases, extensive surgical intervention [5, 6].

In most colorectal centers the management of AL involves the creation of a stoma and endoscopic application of a polyurethan-sponge within the pararectal wound cavity. This use of sponge-assisted EVT for the treatment of anastomotic colorectal leakage has evolved in recent years [7, 8]. This minimally invasive procedure enables continuous drainage, limits the risk of sepsis, promotes granulation and is associated with reduced morbidity, mortality, and hospitalization rates [9]. A significant disadvantage is that in more than 40% of cases, the stoma remains for the rest of the patient’s life.

Sponge-assisted EVT is more suitable for stable patients with early leaks. The earlier the treatment begins, the greater the success rate [7].

However, a major limitation of any sponge-assisted EVT system is that an endoluminal application within the colorectum, either therapeutic or prophylactic, is not feasible because it would occlude the bowel by obstructing the passage. Therefore, this can only work with an established upstream anus praeter. An alternative approach omitting a stoma would be the placement of a covered stent. But this is limited by the lack of drainage of the wound cavity and the high stent migration rate of over 50% [10].

These two major limitations of endoscopic treatment of AL are now overcome by the recently developed VACStent [11]. The VACStent consists of a self-expanding nitinol stent, covered with a silicone membrane and encased in a polyurethane-sponge cylinder, combining the benefits of EVT and covered stents. The ends of the covered stent contact the intestinal wall, sealing it from the lumen. A suction catheter embedded in the open-cell PU sponge is connected to an adjustable vacuum pump. Negative pressure created in the area of the sponge cylinder enables effective drainage as well as strong fixation of the VACStent to the intestinal wall. Covering of the wound cavity and preservation of intestinal passage, drainage and conditioning of the wound surface, and dislocation protection due to the suction cup effect of the vacuum sponge have been demonstrated in initial clinical applications of the upper gastrointestinal tract [12].

This advanced technological principle has now been transferred to the lower GI tract, and initial experience has been gained in this pilot study. The aim of this prospective pilot trial was to investigate the feasibility of the clinical handling of the VACStent, the technical application, safety, and efficacy in patients with anastomotic insufficiency (AI), as well as in patients with high-risk anastomoses and preemptive anastomotic coverage to avoid prophylactic stoma placement.

## Materials and methods

**Fig. 1:**
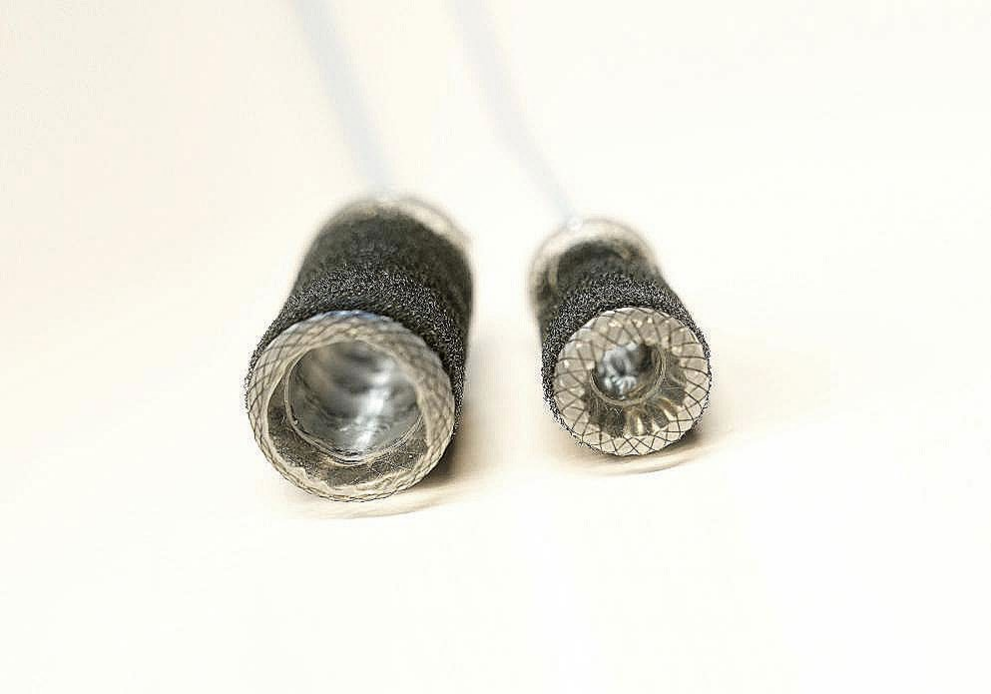
VacStent GI™: left side VACStent Colon (inner diameter 25 mm), right side VACStent Oesophagus (inner diameter 12 mm)

The VACStent (VacStent GI™, VACStent GmbH, Fulda, Germany) consists of a fully covered intestinal stent enclosed by a polyurethane sponge cylinder as described by Lange et al. [13] (Fig. 1).

### VACStent application

After performing transanal endoscopy, a stiff guide wire was placed under direct vision in the descending or transverse colon. The delivery system was then carefully advanced over the wire, and the VACStent deployment was observed via a standard 9 mm gastroscope, which paralleled the delivery system. The application system and guide wire were then removed, the suction catheter was passed through the anus, and connected to a VAC-pump. The continuous suction pressure was  -80 to -125 mmHg. Before removing the VACStent, extensive retrograde rinsing of the sponge via the drainage tube (at least 40 ml 0.9% NaCl) is recommended. Moreover, the suction should be stopped for at least 2 to 4 h before VACStent removal. The device is withdrawn endoscopically with forceps pulling at the retrieval loops placed at the ends of the VACStent. The recommended length of stay for a VACStent was 3 to 7 days.

The EVT Academy provided videos showing the application and removal of the VACStent [14].

### Patients and study strategy

The pilot study was performed by experienced endoscopists at the Cologne Merheim and Holweide Medical tertiary Centers. Ethics approval by the Institutional Review Board (IRB) of Witten/Herdecke University (No. 34/2020) was granted. All patients provided written informed consent to participate in this study. The trial was registered in the clinical trial registry (NCT04884334). Patients were recruited from May 2020 to January 2024.

Three study cohorts were collected and analyzed (Table 1). The trial started with the first two patients, where resections of the sigmoid/rectum were performed together with a protective ileostomy. After AL and large wound cavities were diagnosed, the patients were initially treated with Endo-SPONGEs^®^ (B. Braun, Melsungen, Germany). To finally close the remaining wound cavity, a series of VACStents was applied, analyzing the parameters of technical applicability (implantation, release, suction) and complications (e.g. migration).

**Table 1:** Patient characteristics

Pat. No.	Age, sex	ASA	Relevant risk factors	Disease	Procedure	Localisation of leakage	Days after surgery	Endoscopic treatment before VACStent	VACStent, numbers	VACStent treatment, days	Suction/ pressure mmHg	Complication	Clinical success	Defecation
**Cohort 1**														
1	73, m	3	2x kidney Tx; 3-vessel CHD	Sigmoid perforation in sigmoid diverticulitis with 4-quadrant peritonitis	Sigmoid/rectal resection with primary anastomosis, ileostomy; Dilation of anastomotic stenosis	16 cm, end-to-end anastomosis	13	2x Suprasorb-VAC, 4x Endo-SPONGE^®^	2	10	-100	None	Yes	Ileostomy
2	69, m	3	HIPEC	Appendicial cancer	Surgical cytoreduction and intraoperative HIPEC	11 cm, e-t-e anast.	15	3x Endo-SPONGE^®^	1	5	-125	None	Yes	Ileostomy
**Cohort 2**														
3	62, m	3	COPD	Rectal carcinoma	Lap. anterior rectal resection	6 cm, e-t-e anast.	1	None	4	18	-125	None	Yes	Stool conditioning
4	82, m	3	3-vessel CHD	Sigmoid carcinoma	Lap. sigmoid resection	16 cm, e-t-e anast.	7	None	5	22	-120	Discard of the first VACStent	Yes	Stool conditioning
5	40, m	3	None	Stenosing sigmoid diverticulitis	Sigmoid-rectal resection	12 cm, e-t-e anast.	2	None	2	11	-125	None	Yes	Stool conditioning
6	92, m	3	Left anterior hemiblock, Cor hypertensivum	Colon descendens carcinoma	Left hemicolectomy with subtotal transverse colon resection	14 cm, e-t-e anast.	14	None	2	11	-100	None	Yes	Stool conditioning
7	54, m	2	None	Chronic recurrent diverticulitis	Lap. sigmoid rectum resection en-bloc	8 cm, e-t-e anast.	7	None	3	19	-125	None	Yes	Stool conditioning
8	64, m	3	Steatosis hepatis, COPD	Stenosing sigmoid tumor	Open sigmoid resection with bladder roof and small bowel segment	12 cm, e-t-e anast.	4	None	3	16	-125	None	Yes	Stool conditioning

Pat. No.	Age, sex	ASA	Relevant risk factors	Disease	Procedure	Localisation of anastomosis			VACStent, numbers	VACStent treatment, days	Suction/ pressure mmHg	Complication	AL occurred	Defecation
**Cohort 3**														
9	70, m	3	Parkinson´s disease, bronchial asthma	Mechanical ileus due to sigmoid volvulus, Hartmann-surgery	Open resection of segment rectum and colon descends with descendo-rectostomy, reconnection surgery	9 cm, side-to-end anastomosis			1	7	-125	None	No	Stool conditioning
10	56, m	3	Lung transplantation	Chronic sigmoid diverticulitis with stenosis	Laparoscopic sigmoid/rectal resection	12 cm, e-t-e anast.			1	7	-125	None	No	Stool conditioning
11	61, m	3	Diabetes mellitus Type II, BMI > 30	Decompensated ileus	Laparoscopic sigmoid resection	12 cm, s-t-e anast.			1	7	-125	None	No	Stool conditioning
12	61, m	3	Chronic renal insufficiency, hemodialysis, brain metastases	Recurrent sigmoid diverticulitis	Laparoscopic sigmoid resection	15 cm, e-t-e anast.			1	6	-125	None	No	Stool conditioning

After confirmation of technical success, a further six patients with endoscopically confirmed AL without a stoma were included. They were treated with VACStents, on average, on the 6th day after resection, and the parameters wound coverage, wound healing and fecal passage were analyzed. Stool conditioning was performed by Movicol^®^ (Macrogol) 3 bags/day, and fiber-free nutrition was advised to ensure a soft fecal passage.

A third cohort comprised 4 patients, three with high-risk anastomoses after laparoscopic resection of the sigmoid/rectum with complicated diverticulitis, and one patient with open laparotomy and reanastomosis after Hartmann’s operation. The VACStent was applied intraoperatively in 3 patients, and in one patient at the 1st postoperative day. The primary parameter for this cohort was the analysis of preemptive anastomotic coverage with VACStents to substitute prophylactic stoma placement. A standard anastomosis check was performed on the 7th postoperative day, or earlier if necessary, depending on clinical abnormalities. In the case of clinically ambiguous findings, endoscopy may be supplemented by a CT scan to detect microperforations. Inclusion criteria were endoscopic accessibility of the anastomosis with the introducer catheter of the loaded VACStent and the location of the anastomotic suture line above 5 cm measured from the anal verge. Exclusion criteria were patients with leaks not endoscopically accessible, clinically unstable septic patients, a need for full anticoagulation, or thrombocytopenia < 20.000/µl.

Most operations (*n* = 7) were performed laparoscopically without any conversion to open laparotomy. In 5 patients, a primary open approach was used due to locally advanced tumor situations or complicated diverticulitis involving other organs (bladder, small bowel). All anastomoses were tested intraoperatively by endoscopy and a bubble-test. A pelvic drain placement depended on the course of the operation and the surgeon’s decision, and it only played a role in detecting anastomotic insufficiency in the event of abnormalities. However, routine drainage should be avoided if the surgical site is inconspicuous and the risk profile is low in accordance with the POMGAT guideline. In this study, intraoperative drainage was applied in all 12 patients, which may correlate with the particular risk profile of this patient group.

Postoperatively, an endoscopic anastomotic control was performed no later than the 7th postoperative day, as long as the patient remained clinically stable. Postoperatively, there was a daily check-up for AL observation. If inflammation markers rose (e.g. CRP, Procalcitonin, Leucocytes) and/or clinical signs of sepsis/SIRS occurred, immediate endoscopy was performed, and in unclear situations, an abdominal CT scan. Diagnosis of an AL was defined as either a dehiscence at the suture line, a paracolic wound cavity, or fistula openings.

### Data collection and analysis

Safety, efficacy, and clinical course of the VACStent treatment were analyzed daily from patient enrollment until hospital discharge and during follow-up visits until 6 weeks post-op. All data were collected in a CRF entered into a database, and analyzed. Due to the small number of cases, no statistical analysis was carried out. Only descriptive statistics were used.

### Analysis endpoints

Analysis endpoints were safe practicality, complete leak coverage, effective suction-treatment of anastomoses, and fecal passage. Further endpoints of interest were prevention of septic conditions, successful leak healing, complications, in particular stent-migration, local erosions, bleeding and avoidance of surgical revisions or an anus praeter (Table 2).

**Table 2:** Analyzed parameters and endpoints

VACStent associated parameters (number of VACStents)	Cohort 1 *n* = 3	Cohort 2 *n* = 19	Cohort 3 *n* = 4
Technical application achieved	*n* = 3	*n* = 19	*n* = 4
Continuous suction	*n* = 3	*n* = 19	*n* = 4
Migration	*n* = 0	*n* = 2	*n* = 0
Clogged sponge	*n* = 0	*n* = 0	*n* = 0
Local bleeding	*n* = 0	*n* = 0	*n* = 0
Discomfort in the small pelvis	*n* = 0	*n* = 1	*n* = 1
Transmural ulcer	*n* = 0	*n* = 0	*n* = 0
Fecal flow	*n* = 0	*n* = 6	*n* = 4
VACStent removal	*n* = 3	*n* = 19	*n* = 4

Clinical endpoints (number of patients)	Cohort 1 *n* = 2	Cohort 2 *n* = 6	Cohort 3 *n* = 4
AL closure	*n* = 2	*n* = 6	/
Surgical revision	*n* = 0	*n* = 0	*n* = 0
Stoma creation	/	*n* = 0	*n* = 0
Anastomotic stenosis	*n* = 0	*n* = 0	*n* = 0
Sepsis/SIRS	*n* = 0	*n* = 0	*n* = 0

## Results

### Initial patient cohort with anus praeter and AL

The first two patients already had an established ileostomy and larger wound cavities. After endocavitary sponge treatment, the idea was to support wound closure by the VACStent.

1. Patient: The first patient was a 73-year-old high-risk diverticulitis patient (immunosuppressed due to a kidney transplant) who underwent open laparotomy for peritonitis, sigmoid/rectum resection for perforated diverticulitis along with an ileostomy. After endoscopic dilatation of an anastomotic stenosis, an AL with a large wound cavity developed, which was treated with Suprasorb^®^-sponges twice and Endo-SPONGE^®^ four times over 22 days. The treatment was continued with the VACStent for 10 days, and the cavity closed except a minimal residual. Follow-up after three months was unremarkable.
2. Patient: The second patient was a 69-year-old diagnosed with appendiceal cancer, treated with extended surgical cytoreduction and intraoperative hyperthermic chemoperfusion (HIPEC). The sigmoid/rectum was removed, and descendo-rectostomy was performed along with a protective ileostomy. This anastomosis developed an AL with a pararectal wound cavity. The AL was treated for 15 days with three consecutive Endo-SPONGEs^®^, followed by a 5 day VACStent treatment. The AL healed completely, despite a small remaining fistula ostium.

### Second patient cohort with no previous endoscopic treatment of the AL and without a diverting stoma

**Fig. 2:**
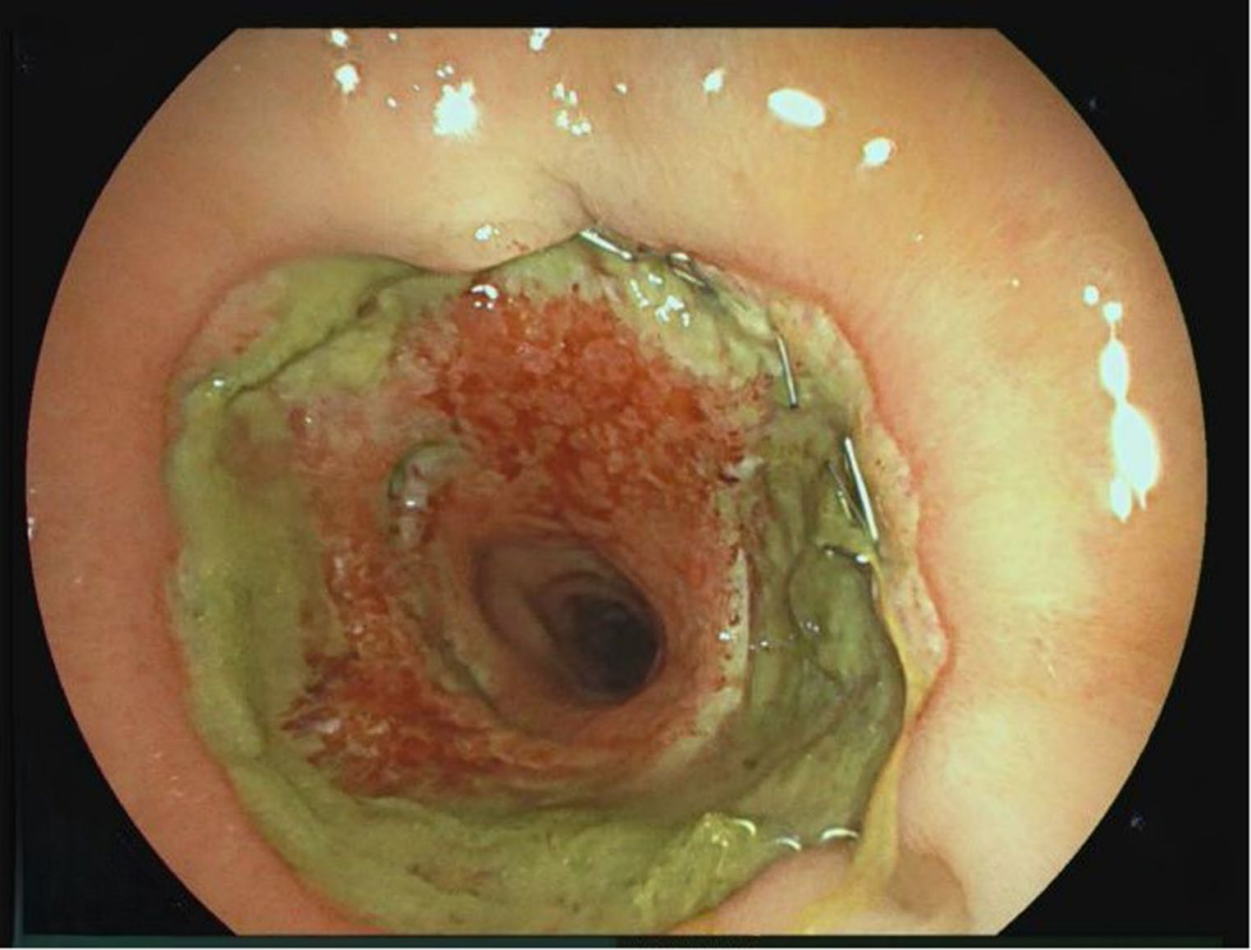
Circular fibrin layer at the anastomosis as AL precursor

**Fig. 3:**
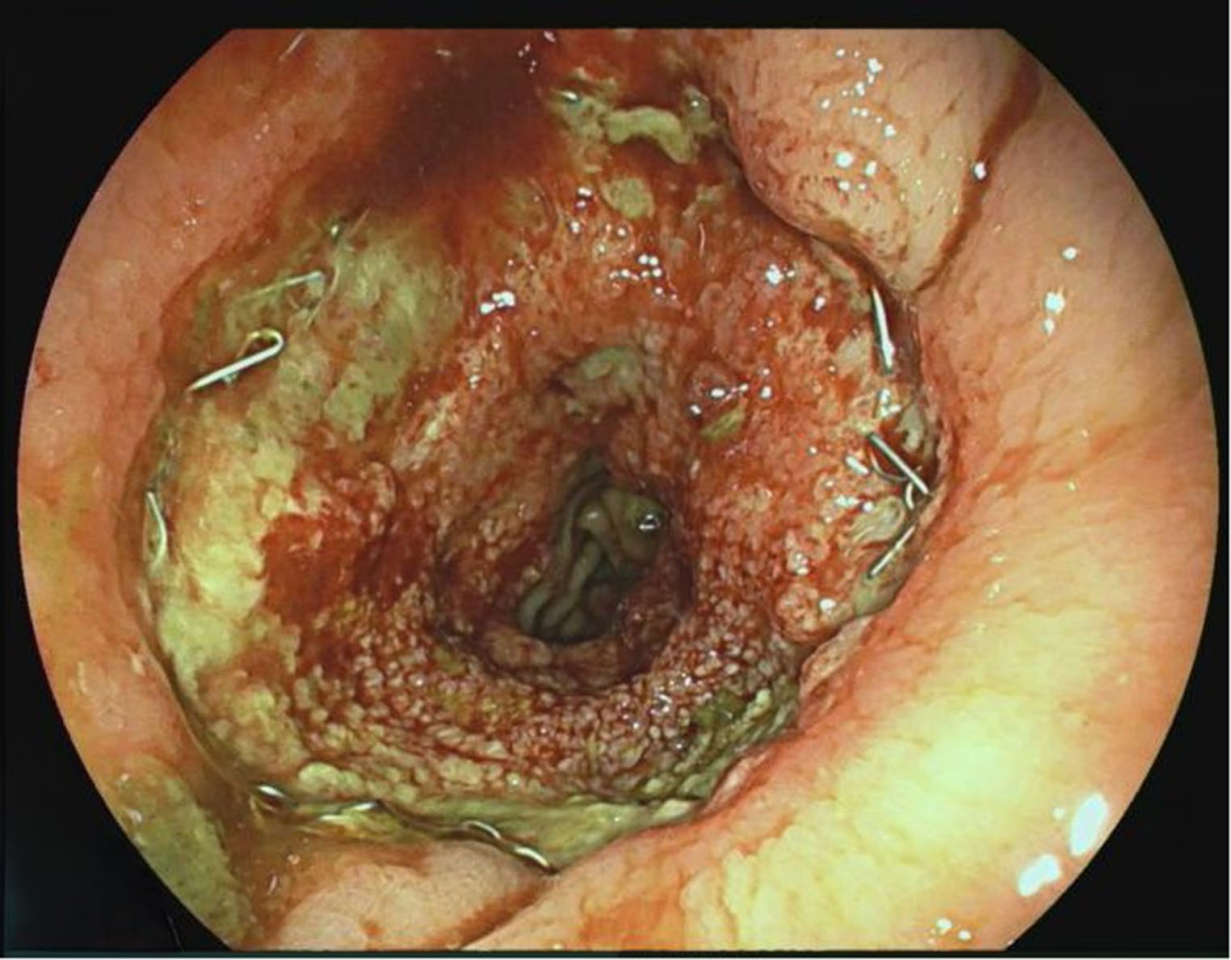
AL with small transmural leakage

**Fig. 4:**
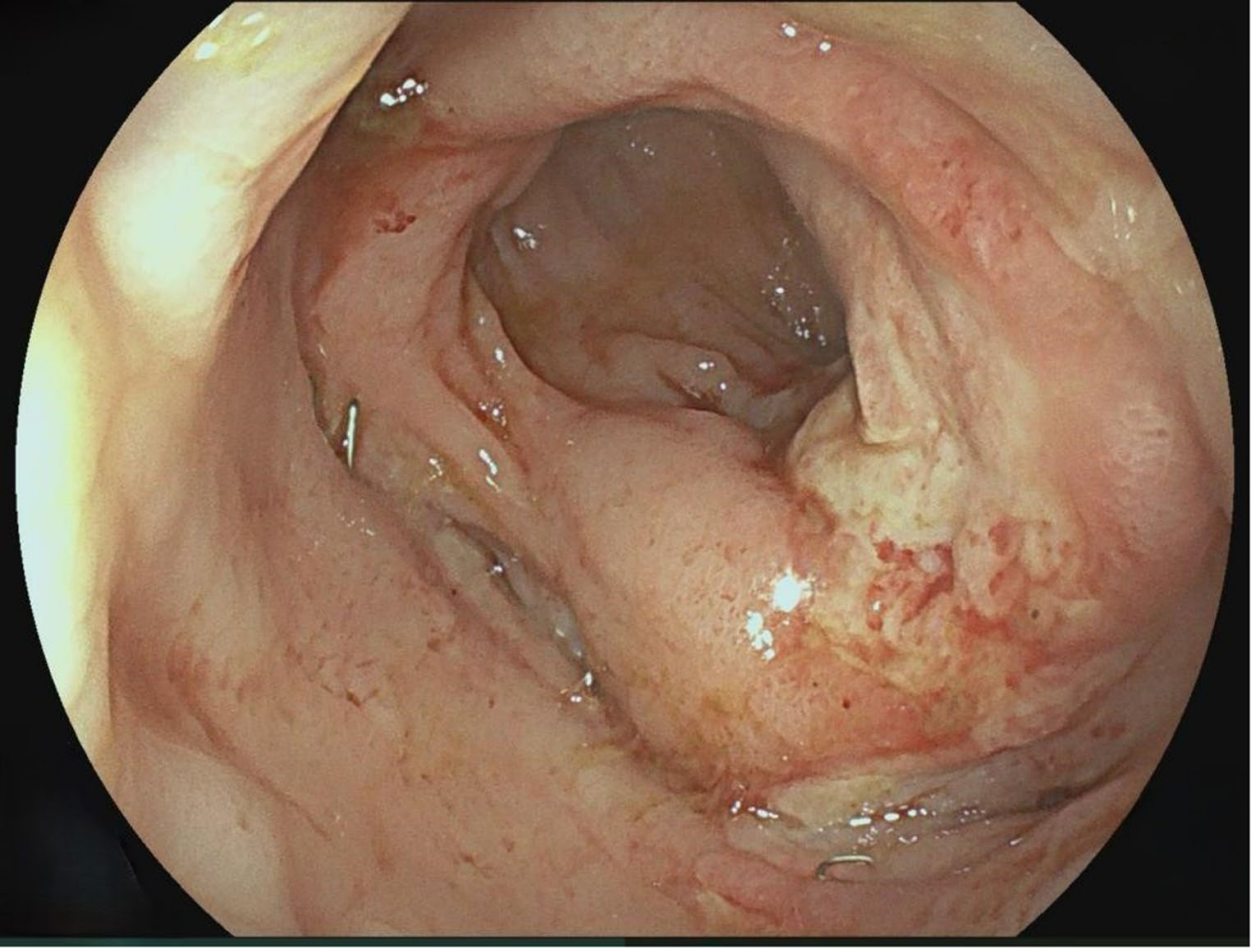
Healed anastomosis after treatment with 5 VACStents for 22 days

**Fig. 5:**
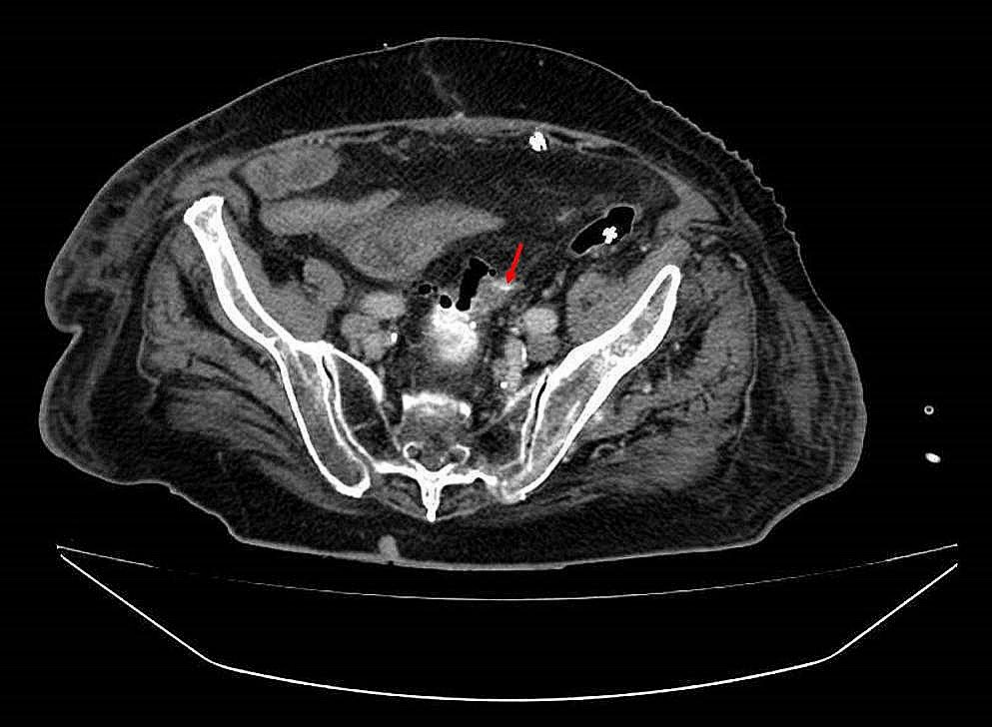
Abdominal CT of AL with rectal contrast medium leakage (red arrow)

**Fig. 6:**
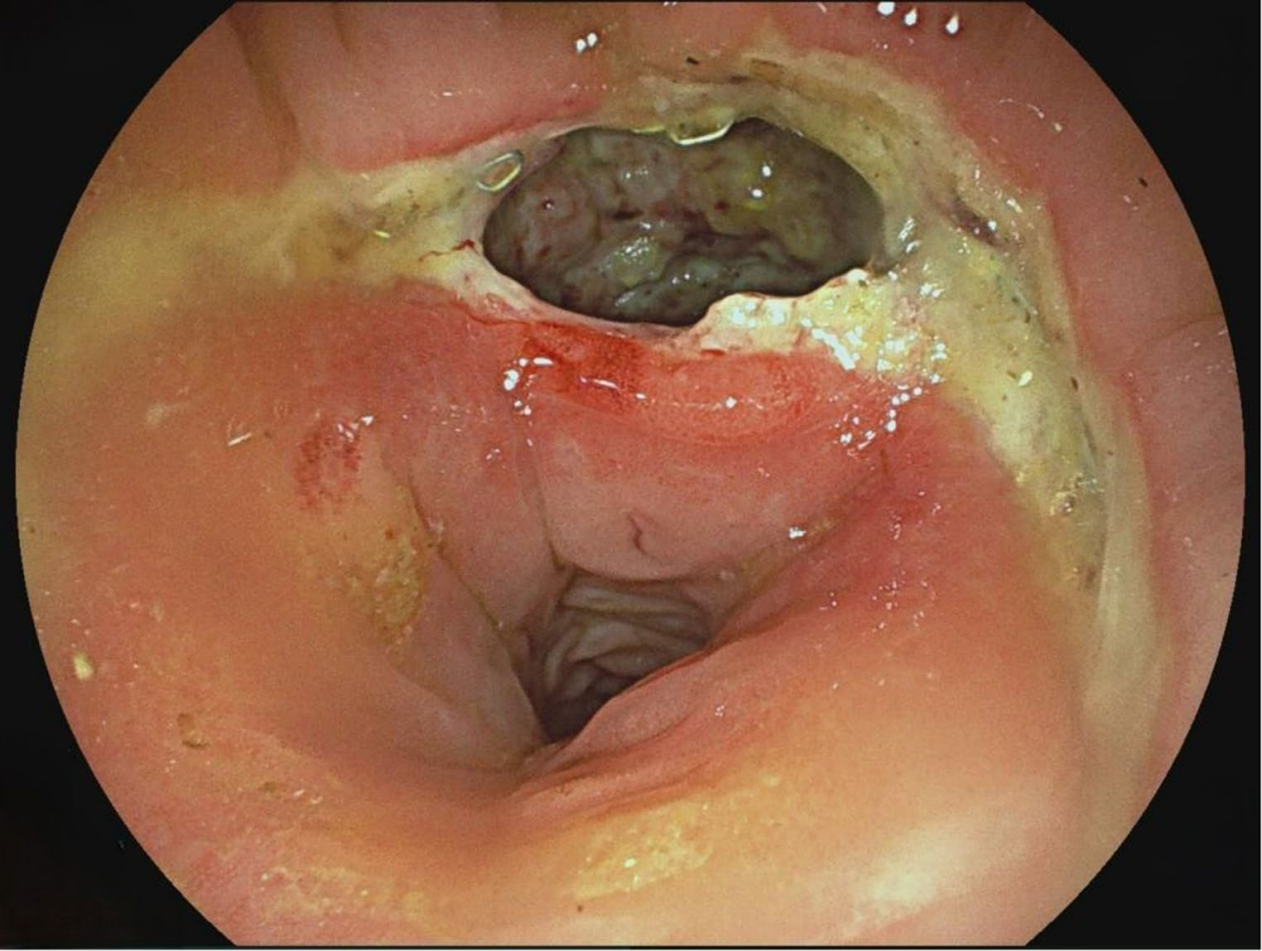
AL with large pararectal wound cavity

3. Patient: A 62-year-old patient (ASA 3) with a rectal carcinoma underwent laparoscopic anterior resection and an anastomosis was created at 6 cm from the anal verge. Endoscopy was performed on the first postoperative day due to color changes in the pelvic drainage secretion. A circular fibrin layer with poorly perfused mucosal areas was found at the anastomosis, and a VACStent was applied (Fig. 2). The VACStent was changed after 3 days and then removed after a further 4 days. However, 3 days later, an AL with small transmural leakage appeared, which healed after 11 days with renewed treatment by two VACStents (Fig. 3).

4. Patient: After laparoscopic sigmoid/rectal resection for sigmoid carcinoma, an 82-year-old high-risk patient developed AL, which was diagnosed in the control endoscopy on day 7 and treated with a VACStent. Three days later, the stent dislocated and was discarded. After another 4 VACStents over 20 days without complications, the anastomosis healed completely (Fig. 4).

5. Patient: A 40-year-old patient (ASA 3) suffering from relapsing stenosing sigmoid diverticulitis was treated with laparoscopic sigmoid–rectal resection. Due to clinical deterioration, abdominal CT on day 2 showed evidence of AL, which was initially not detected endoscopically. During the laparoscopic surgical revision, the abdomen was flushed, drained, and a VACStent was implanted. After a further VACStent change after 4 days and a total treatment of 11 days, the AL healed completely.
6. Patient: Due to a bleeding descending colon carcinoma, an extended left hemicolectomy with subtotal transverse colon resection and transverso-rectostomy was performed in a 92-year-old patient. In case of clinical deterioration, AL was detected in the abdominal CT by rectal contrast medium leakage (Fig. 5, red arrow) on the 14th day. Treatment was initially carried out with the VACStent for 6 days and then, after changing, for a total of 11 days until healing of the AL.

7. Patient: A laparoscopic sigmoid rectum resection was performed for chronic recurrent complicated (ileum fistula, retrovesical abscess) perforated diverticulitis. On day 7, the control endoscopy showed an AL with a wound cavity pararectally (Fig. 6). After a total of 3 VACStents over 19 days, the AL healed well.

8. Patient: A 64-year-old patient (ASA 3, CHD, aortic aneurysm) with a stenosing sigmoid conglomerate tumor in the small pelvis underwent open sigmoid resection with removal of the bladder roof and an ileum loop. On the 4th postoperative day, AL was detected endoscopically with stool-like drainage secretions and treated with 3 VACStents. After a total of 16 days indwelling time, the AL healed completely.

At least one follow-up visit was performed in all patients, varying from one week to 6 months without findings.

### Third cohort with prophylactic VACStent application in high-risk anastomoses to substitute protective stoma placement

In this cohort, the intraoperative application of the VACStent directly after surgical creation of the anastomosis was tested. The aim of this approach was to avoid prophylactic stoma creation.

**Fig. 7:**
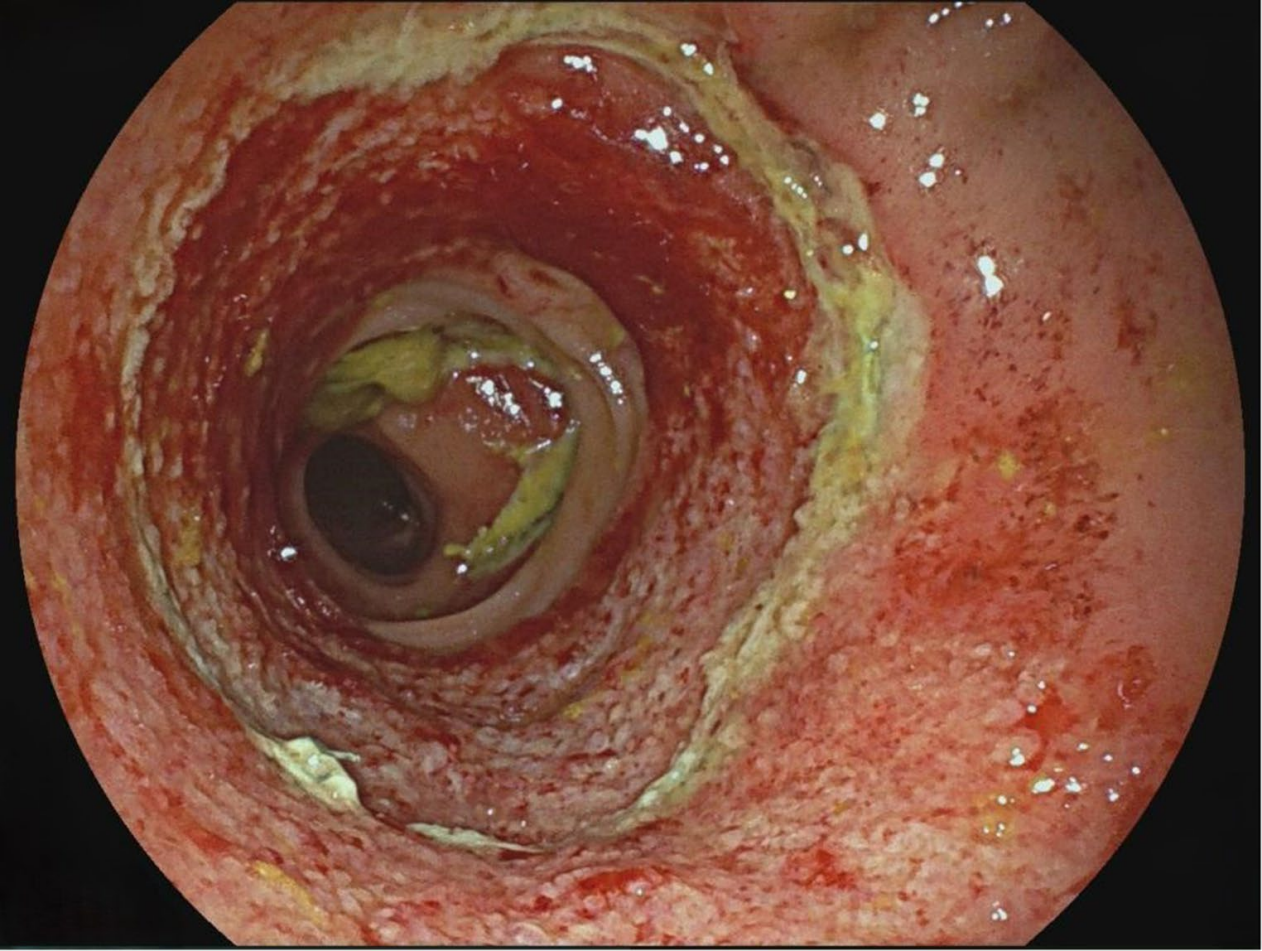
Healing anastomosis after 7 days with distinct granulations

**Fig. 8:**
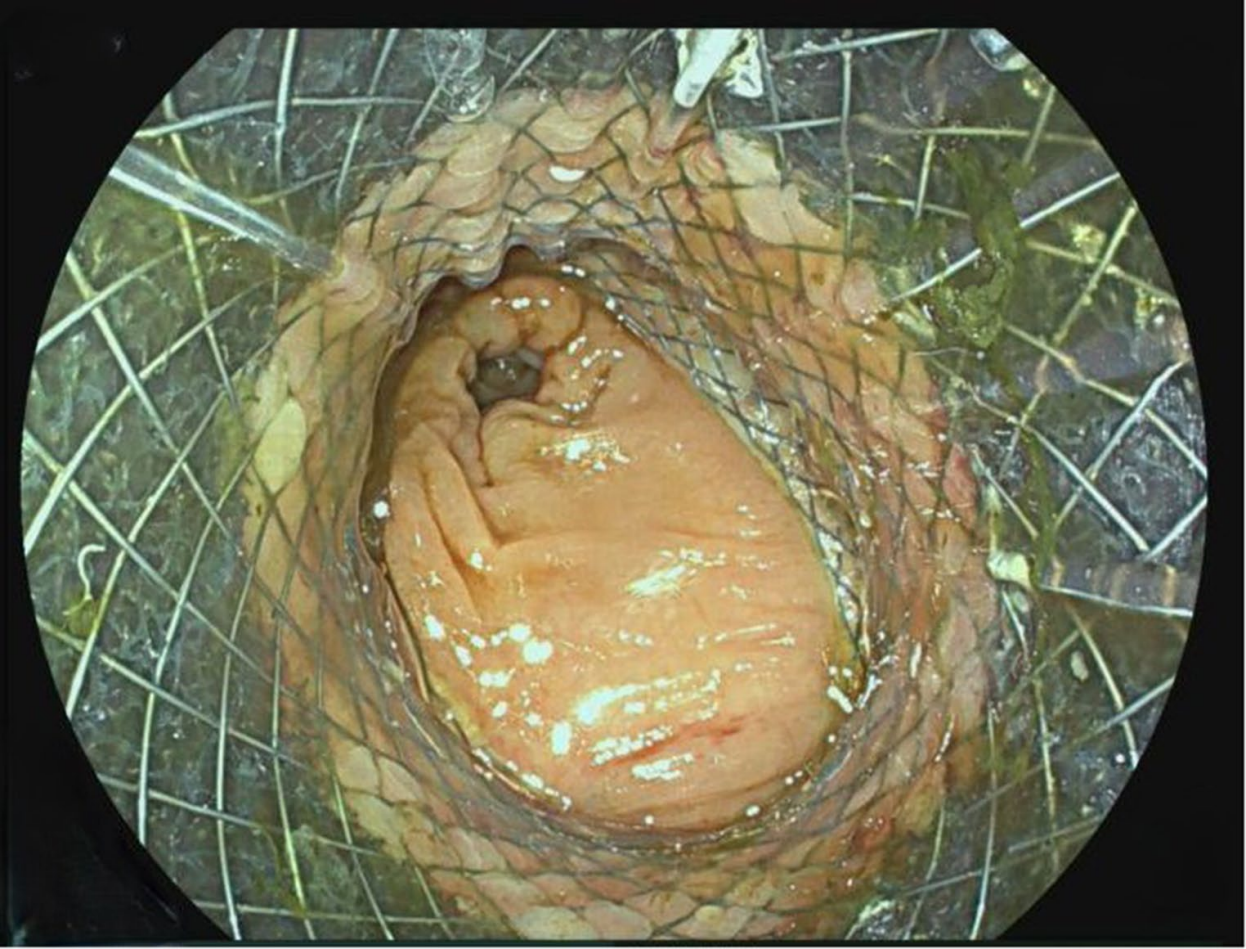
Free passage through the VACStent by a coloscope

9. Patient: A 70-year-old patient (Parkinson’s disease, bronchial asthma) underwent reconnection surgery between the colon descendens and the upper part of the rectum as a descendo-rectostomy. This was preceded by a discontinuity resection for ileus caused by a sigmoid volvulus with a terminal colostomy and rectal occlusion (Hartmann-procedure). To avoid a new stoma, the VACStent was applied intraoperatively for 7 days, which showed problem-free healing of the anastomosis after removal.
10. Patient: A 56-year-old patient with a lung transplant had to undergo laparoscopic sigmoid resection due to chronic recurrent diverticulitis leading to stenosis. The necessary immunosuppression for rejection control required either a protective stoma or a preemptive VACStent application. The VACStent was implanted for 7 days and then removed without any problems, resulting in a well-healed anastomosis (Fig. 7).

11. Patient: Due to a decompensated ileus with chronic recurrent acute phlegmonous diverticulitis, a 61-year-old patient underwent laparoscopic sigmoid resection. Due to the dilated colon, an end-to-side recto-descendostomy was performed and a VACStent was applied on the 1st postoperative day due to impaired mucosal perfusion. After 7 days, the VACStent was removed, and the anastomosis healed well without any subsequent problems.
12. Patient: A 61-year-old patient with metastatic adenocarcinoma of the gastro-esophageal junction and brain metastases, who had undergone stereotactic radiotherapy, developed increasingly aggravated recurrent sigmoid diverticulitis during chemotherapy. This was then resected laparoscopically as a sigmoid resection under ongoing dexamethasone therapy, and a VACStent was applied intraoperatively for 7 days (Fig. 8). After removal, the anastomosis healed completely without complications.

All of these patients had follow-up visits two to six weeks after hospital discharge without findings.

### Clinical endpoints

The safe and easy applications of the VACStent in the lower GI tract was demonstrated in all 12 cases. VACStent-associated complications such as clinically relevant ulcers, bleeding or VACStent displacement did not occur. However in two cases, migration was observed with the 12 mm free inside diameter VACStent, but never with the 25 mm diameter version (VACStent-Colon).

These VACStents with 12 mm diameter were applied in 5 patients. In 3 patients with AL without an anus praeter, good healing but functional narrowing in the stent area (12 mm free diameter) exhibiting slow stool passage was observed. Overall, one VACStent was expelled, and one showed minor migration. Subsequent implantation of the size-matched VACStent-Colon (25 mm free diameter) in 7 patients resulted in good unrestricted stool passage without migration due to colon peristalsis.

The continuous suction pressure was a median of -125 mmHg (-80 to -125 mmHg), and the median length of stay with VACStents was 13.5 days. The median indwelling time per VACStent was 6 days. All patients were characterized by relevant risk factors, as reflected by the ASA 3 score (American Society of Anesthesiologists) in 11 of 12 patients, grade 2 obesity (BMI > 30) or severe secondary diseases.

Exploratory analysis of endpoints showed that safe practicality, complete leak coverage, effective suction-treatment of anastomoses, and fecal passage were successfully achieved for all patients. Clinical endpoints such as prevention of septic conditions, successful leak healing, complications (in particular stent-migration, local erosions, bleeding), and avoidance of a stoma were also achieved without limiting findings (Table 2).

## Discussion

AL remains the most problematic complication in colorectal surgery and has significant negative consequences with increased morbidity and mortality. The creation of a deviating stoma is central to complication management to counteract the development of sepsis. The AL itself is not directly affected by the stoma. However, a stoma is not only used therapeutically in diagnosed AL but also prophylactically in high-risk constellations. Such a stoma is not only a major problem for the patient but also has considerable complications. In addition to local problems such as stoma prolapse, stoma stenosis, skin irritation, and fistula formation, reconnection of the stoma requires further surgery with a new surgical suture line and long-term consequences due to intestinal stenosis, adhesions, and incisional hernias [15].

The current standard of endoscopic AL treatment is sponge-assisted EVT as intracavitary therapy of the AL-associated pararectal wound cavity. The Endo-SPONGE^®^ is available as a commercial product and shows very good clinical results. However, it is usually necessary to create a stoma, as clogging of the sponge by the aspirated stool in particular can severely impair functionality. The endoluminal application of the Endo-SPONGE^®^ is not possible without a diverting stoma, as the colon is closed and blocked by the suction.

For the first time, the VACStent now represents a reasonable clinical alternative for the treatment of AL without the need to create a stoma. This means that the VACStent must not only ensure wound closure and drainage of wound secretions but also the free passage of stool.

These facts also have an impact on cost-effectiveness, as the number of endoscopies required is reduced from an average of 6–9 with the Endo-SPONGE^®^ to 3, with a more than doubled lay time of 7 compared to 2 to 4 days. Despite the significantly higher costs for the VACStent, this could result in comparable treatment costs overall. If the direct and follow-up costs of the stoma creation and reconnection surgery are also taken into account, this probably results in a considerable improvement in cost-effectiveness.

The VACStent was initially developed for the upper GI tract and showed convincing results in the handling and treatment of esophageal leaks. In particular, the fact that the patient could swallow well with the VACStent in place and oral food could be built up without fear of migration suggested that this concept should also be applied to colorectal surgery.

Until now, two different endoscopic techniques have been used to treat AL in colorectal surgery: the covered stent and the sponge associated EVT. The covered stent has not been established due to the lack of drainage function and the high rate of dislocation. Intraluminal sponge EVT has the disadvantage of requiring a deviating stoma, as the suction leads to occlusion of the colon. As a method for treating a wound cavity, sponge EVT has set new standards and has shown very good results. With the development of the VACStent, it has now been possible to combine the advantages of the stent - direct wound closure and fecal flow - with the suction and drainage function of sponge EVT. This ensures a very good NPWT effect on the anastomosis and an open passage of the positionally stable VACStent.

Taking the clinical evidence together, transferring the positive clinical experience of the upper GI to lower gastrointestinal lesions using the VACStent was the next development step. In this pilot study, the first two patients were treated initially with an ileostoma and endocavitary sponges, and afterwards the residual cavities were sealed with VACStents. This first cohort proved the easy and safe applicability, the position-stable localization without migration, and effective negative pressure wound treatment.

Having proved the technical questions, the second patient cohort was collected, with no previous treatment of the leakage and no deviating stoma in place. The fecal passage was influenced by the free diameter of the VACStent. The standard VACStent has a free 12 mm diameter, which has limitations if the stool thickens. Clinically this was indicated by abdominal distension and discomfort. Therefore, a new design variant, the Colon-VACStent, was manufactured, which has a 25 mm free diameter allowing easy passage of the stool. However, softening of the stool by Macrogol and fiber-free diet was recommended in all cases. It is impressive to watch the patient moving around at the ward using the normal toilet for defecation with the VACStent in place.

Finally, a third patient cohort was initialized, to prove the usability of the VACStent for a preemptive intraoperative application to substitute for an otherwise prophylactic stoma in high-risk patients. The experience of a pilot study in the upper GI to reduce AL using preemptive EVT provided the rationale [16]. Even if suture leakage occurred under preemptive EVT, the suction drainage of the sponge cylinder effectively prevented the formation of a larger wound cavity and the development of sepsis [16].

The limitations of this study are the limited number of cases of 26 VACStent procedures, which so far only allow valid statements on manageability and safety. However, the results in the various indications give a clear indication of the potential of the VACStent in colorectal surgery. To date, the VACStent has not been used below a distance of 4 cm from the linea dentata of the anus to avoid discomfort in the small pelvis caused by the VACStent touching the pelvic floor or anal sphincter. In the future, a further modification of the VACStent will be able to specifically address the deep anastomoses in the lower rectum.

## Conclusion

According to this first clinical pilot study, anastomotic colonic leaks can be treated successfully by the VACStent potentially replacing the need of an anus praeter in colorectal resections. This might cause a paradigm shift in colorectal complication management. The traditional principle of aggressive surgical therapy plus drainage might be replaced by this endoscopic technique in the future. The clinical development of VACStent-induced EVT and the development of broad spectrum indications have just started.

## Data Availability

The raw data supporting the conclusions of this article will be made available by the authors, without undue reservation.
